# Esports and Visual Attention: Evaluating In-Game Advertising through Eye-Tracking during the Game Viewing Experience

**DOI:** 10.3390/brainsci12101345

**Published:** 2022-10-04

**Authors:** Marco Mancini, Patrizia Cherubino, Giulia Cartocci, Ana Martinez, Gianluca Di Flumeri, Luca Petruzzellis, Michele Cimini, Pietro Aricò, Arianna Trettel, Fabio Babiloni

**Affiliations:** 1 BrainSigns Srl, Via Lungotevere Michelangelo 9, 00192 Rome, Italy; 2 Faculty of Economics, UNINT—Università degli Studi Internazionali di Roma, Via delle Sette Chiese 139, 00147 Rome, Italy; 3 Department of Molecular Medicine, Sapienza University of Rome, Viale Regina Elena 291, 00161 Rome, Italy; 4 Department of Communication and Social Research, Sapienza University of Rome, Via Salaria 113, 00198 Rome, Italy; 5 Department of Physics, University of Bari Aldo Moro, Via Orabona 4, 70125 Bari, Italy; 6 Department of Computer, Control and Management Engineering “Antonio Ruberti”, Sapienza University of Rome, 00185 Rome, Italy; 7 College of Computer Science and Technology, Hangzhou Dianzi University, Hangzhou 310005, China

**Keywords:** eye tracking, visual attention, esports, in-game advertising (IGA), Twitch, advertising, gaming

## Abstract

In recent years, technological advances and the introduction of social streaming platforms (e.g., Twitch) have contributed to an increase in the popularity of esports, a highly profitable industry with millions of active users. In this context, there is little evidence, if any, on how users perceive in-game advertising (IGA) and other key elements of the game viewing experience (e.g., facecam and chat) in terms of visual attention. The present eye-tracking study aimed at investigating those aspects, and introducing an eye-tracking research protocol specifically designed to accurately measure the visual attention associated with key elements of the game viewing experience. Results showed that (1) the ads available in the game view (IGAs) are capable altogether to attract 3.49% of the users’ visual attention; (2) the chat section draws 10.68% of the users’ visual attention and more than the streamer’s face, known as a powerful attentional driver; (3) the animated ad format elicits higher visual attention (1.46%) than the static format (1.12%); and (4) in some circumstances, the visual attention elicited by the ads is higher in the “Goal” scenes (0.69%) in comparison to “No-Goal” scenes (0.51%). Relevant managerial implications and future directions for the esports industry are reported and discussed.

## 1. Introduction

Around the late 1990s and early 2000s, while the popularity of video games soared thanks to the proliferation of competitions often sponsored by Intel and AMD, new business models for the gaming industry emerged [1]. Red Annihilation (1997), a tournament based on the video game *Quake*, was the first esports experience capable to draw over 2000 participants and paved the way a few years later for the introduction of a new term, “eSports”, coined during the inauguration of the Online Gamers Association [2,3].

In today’s digital youth culture, esports refers to an area of sports activities in which online users employ information and communication technologies to improve their mental or physical abilities through competitive computer gaming [2].

Twitch, Facebook Gaming, and YouTube Gaming are the most well-known streaming platforms, handling the process of transmitting video games in real-time over a live broadcast platform and allowing the interaction between those who participate in the game and those who watch and communicate with the streamer or other viewers [4]. Such platforms meet the users’ need to compete in tournaments, be a part of the gaming community, watch their favorite players and teams, reconnect with friends, and meet professional gamers [5].

Although China is currently the leading country with 530.4 million esports-aware people, audience and awareness numbers are also rising exponentially in emerging markets such as Latin America, the Middle East and Africa, and Southeast Asia. Behind this success, a key role is played by the esports-related revenue, related to six main sources: “Sponsorship”, “Media rights”, “Merchandise & tickets”, “Publisher fees”, “Digital”, and “Streaming.” In particular, sponsorship, which represents the highest source of revenue, refers to the revenues generated by teams and organizers through sponsorship deals, where the advertisements, sold as part of a sponsorship package, have a significant impact [6]. This type of revenue allows players, coaches, and staff in top teams to regularly receive a salary in return for their performance publicly shown on the mainstreaming platforms during their short professional gaming career (2 years and until 23 years old, on average) [7]. Some authors have aimed to shed light on the reasons that lead people to watch others play video games, referring that the live-broadcasting nature of video game streaming, as a major new force in the games industry, creates a unique interaction between media creator and media consumer, generating new opportunity to learn and be updated on popular games [8,9,10].

### Attention and In-Game Advertising

Esports represents a great opportunity for advertisers [11] since players generally perceive ads in video games as a positive experience if they do not interrupt the game itself [12,13]. The advertisers’ approach to integrating commercial messages into noncommercial or entertaining media recalls the widely studied technique of “product placement”, where brands and products are presented to an engaged audience as components of the normal flow of a movie, television show, or other forms of entertainment [14,15,16]. In this direction, it has been shown that if the brand is associated with a popular show, song, or influencer [17,18], it generates a 20% increase in brand awareness [16]. Another reason underlying the fact that gamers are increasingly willing to accept the product placement strategy is represented by its crucial role in saving video game production costs and, consequently, video game market prices [19]. In addition, the relationship between the streamer and the sponsorship helps to convey the product value with no need for additional words and leads to a deeper emotional connection with the brand [20,21]. Since users tend to ignore any kind of advertising [22,23,24,25], even blocking advertising on their devices to avoid tracking their online behavior, the practice of product placement seems to be the right strategy to engage the audience, especially concerning millennials and Generation Z [26]. From an ethical perspective, it is worth noting that such strategies are seen by a part of the scientific community as a form of media exploitation and an intrusion into the privacy of viewers, aimed to influence targets such as millennials and Generation Z that have been demonstrated as harder to manipulate in comparison to the other generations [19,21].

In this context, in-game advertising (IGA), described as “the integration of non-fictional products and brands within the playing environment of video and computer games through simulated real-life marketing communication mechanisms” [27], is a unique and highly profitable contact point between the streamer and the sponsorship [28].

However, there is still a lack of evidence on how online users perceive advertisements on these streaming platforms that combine traditional social media elements and entertainment [29], and on the capability of in-game ads to properly draw the users’ attention [30]. Attention, defined as the allocation of mental, visual, or cognitive resources to an object of interest, is strictly required for any communication message that aims to be effective [31]. Understanding the consumers’ cognitive processes in terms of the attention can assist experts in the attempt to effectively orient advertising and marketing activities using the eye tracker [32].

Despite the exponential growth of the esports industry and advertising-related revenues, most of the eye-tracking studies conducted in gaming contexts focused on game-based learning [33,34], game design [35,36], and the understanding of in-game engagement [37], leaving the mechanisms that play an important role in conveying the users’ attention toward IGAs essentially unexplored [30].

In contrast, eye-tracking research aims to shed more light on attentional processes in the traditional sports industry is relatively more common. For example, Oboudi et al. (2022) [38] investigated differences in attention based on the attractiveness of the game and the color of prosocial messages, finding that both factors significantly affected the visual attention elicited by prosocial messages. Colors, as shown by the eye-tracking study conducted by Toh et al. (2022) [39] in the context of sports sponsorship, can be also effectively manipulated to affect visual attention toward logos. Regarding sponsorship in sports event posters, Alonso Dos Santos et al. (2019) [40] showed through their study that sponsors capable of drawing only 2.8% of the total exposure time (sponsorship blindness) and that sponsors positioned in the poster’s region of action elicit more attention than the others. Other cues about the relevance of sponsor positioning have been provided by Breuer and Rumpf (2012) [41], who investigated visual attention toward sponsorship in sports telecasts through eye tracking, revealing that visual attention is affected by the positioning of sponsor signs. In the digital context, an eye-tracking study [42] conducted on the Vancouver 2010 Olympic Games website reported that usability factors such as size, positioning, and clickable elements contribute to increasing the visual attention elicited by the related areas of interest (AOIs). In addition, the authors found that women and expert online buyers were less attracted by the site’s sports-related components and more concerned about the shopping activity itself.

The investigation of attention in the gaming context represents a challenge, especially if peripheral or implicit [43,44], given that the player’s limited attention is already taken by the game [45]. Interestingly, a recent eye-tracking study aimed to assess the effectiveness of esports sponsorship showed how the ad’s exposure time, a widely accepted metric employed to evaluate the effectiveness of the ads, reported an error of 60.18% concerning the real visual experience [46]. Such a result highlights the importance of employing eye tracker technology to objectively measure the visual attention of a group of users while dealing with ads during the game viewing experience. In addition, this latter is also featured by the presence of other two key elements, represented by the chat and the facecam (streamer’s face). Researchers have been studied the chat component for decades, with the earliest studies reaching as far back as the 1970s [47,48]. Within Twitch’s interface, an interactive and real-time chat area, playing a primary role in terms of social communication and entertainment, is shown prominently on the side of the stream and can be used by both the audience and the streamer [49]. The facecam, or the streamer’s area, located in the top-right position of Twitch’s interface in a webcam overlay, is another key element that contributes to increasing social connectivity and interactivity and assists the streamer in positioning his/herself as a public person or a micro-celebrity that take care of self-branding [28,50,51]. In this context, the current lack of information on how much attention users pay to the ads and other crucial elements of the game viewing experience [30] calls for the investigation of such aspects of interest (see RQ1).

Focusing on the effectiveness of animated vs. static ads, current literature has shown controversial results concerning visual attention. Although it has been found that animation increases the visual attention toward the ad [52,53,54,55], some authors [56] believe that animated ads, being more noticeable, are more likely to be ignored, thus not helping advertisers to reduce the banner blindness phenomenon [57]. In this direction, it has been found that animated ads attract less attention, especially after long exposure, than static ads [58], animated ads capture the users’ attention significantly more than static banners only if a human face is included in the ad [59], or no differences have been found at all in the comparison of the visual attention elicited by animated and static ads [60]. For these reasons, it would be crucial to assess if animated banner ads are more effective than static banner ads in drawing the users’ visual attention concerning the game viewing experience (see RQ2).

According to the limited-capacity model of attention [61,62], the attentional human capacity is limited, and if the total attentional capacity is allocated to a primary task, less attention or no attention at all will be given to the secondary task [63]. In the same way, esports spectators are less likely to pay attention to ads (secondary task) when the game itself (primary task) becomes more engaging [62,64]. Seo et al. (2018) [30] applied the limited-capacity model to esports while investigating the amount of attention captured by ads in battle and non-battle scenes of a popular game (StarCraft). The authors found that participants’ attention toward the ads was significantly higher during the vision of “non-battle” scenes in comparison to “battle” scenes. Beyond traditional war games, the “battle” concept also plays a key role in soccer games, often described using terms directly associated with the battlefield and where the “war metaphor” is highly salient [65,66]. Although scenes featured by goal-scoring are the highlights for most audiences, representing the most emotional and highly engaging scenes of a soccer match [67], they also include moments (i.e., celebration after scoring) that completely break or interrupt the battle on the soccer field. For this reason, during the “Goal” scenes, the allocation of the attentional resources could shift more likely from the primary task (the game itself) to the secondary task (ads), in comparison to scenes (“No-Goal”) where the battle is not interrupted by any significant event. In this context, it would be important to verify if the amount of visual attention captured by the ads is equally distributed across “Goal” and “No-Goal” scenes (RQ3).

According to the aforementioned aspects, this study aimed to answer the following research questions:

- RQ1: How much visual attention does IGA draw and how different is it from that captured by other important elements (chat and facecam) of the game viewing experience?
- RQ2: Concerning the game viewing experience, are animated banner ads more effective than static banner ads in drawing the users’ visual attention?
- RQ3: Is the amount of visual attention captured by the ads equally distributed across “Goal” and “No-Goal” scenes?

## 2. Materials and Methods

### 2.1. Participants

The study involved 47 male participants with a mean age of 23 years old (SD = 4.1 years). The recruitment aimed to obtain a sample featured by the prevalent demographics of those who watch live gaming streams on Twitch, considering that users between 16 and 34 years old account for 72% [68] and males for the 78% [69] of viewers. Participants signed an informed consent after being properly informed and instructed about the study. The experiment was carried out under the principles outlined in the Declaration of Helsinki of 1975, as revised in 2008, and it was approved by the Sapienza University of Rome Ethical Committee in charge of the Department of Molecular Medicine.

### 2.2. Experimental Protocol

Before each trial, participants were requested to sit comfortably at a workstation in front of a 22-inch screen, under which an eye tracker was properly placed to record the participants’ eye movements during the entire experimental session. Then, a short period of the eye-tracking calibration was employed to estimate the geometric characteristics of each subject’s eyes and guarantee an accurate gaze point calculation [70]. Once the calibration process was successfully performed, participants received the following instruction: “A video game enthusiast friend of yours informed you about Twitch, a platform where you can watch the FIFA20 champions play in real time. We ask you to naturally watch some videos related to a FIFA20 match that was recently streamed through the Twitch platform”.

Afterward, participants were exposed to 4 videos, lasting 3 min each, all related to the same FIFA20 match, which involved live streaming hosted on Twitch by a famous Italian pro player.

Each video included also the facecam (the streamer’s area), the chat, and the IGAs. These latter included two banners respectively located in the bottom-left (“Left Banner”) and the bottom-right position (“Right Banner”), and the digital billboards (“Digital Billboards”), whose position changed according to the framing of the match. The brands shown by the “Left Banner” and the “Digital Billboards” included global brands related to clothing, electronics, banking, automotive, aviation, food and beverage, and real estate. The “Right Banner” included one of the best-known Italian brands linked to the sports media industry. The total video exposure lasted 12 min (3 min per video), 2 min less than the original FIFA20 match (14 min in total). Such a cut was performed to ensure the same duration for all 4 videos of interest, each of those included “Goal” or “No-Goal” scenes and “Static” or “Animated” ads, resulting in 4 videos labeled as follows: “No-Goal/Static Ads”, “Goal/Static Ads”, “No-Goal/Animated Ads”, and “Goal/Animated Ads.” It is important to point out that the classification “Static/Animated” was performed exclusively taking into account the nature of the “Left Banner”, which was the only ad that was presented in both static and animated format. See Appendix A for more details about the stimuli employed.

Each participant was exposed to all 4 videos, presented in a random sequence generated through a computer-based randomization procedure (“Research Randomizer” tool, available at the following link: https://www.randomizer.org (accessed on 14 January 2022). 

### 2.3. Data Recording and Signal Processing

The eye-tracking data were recorded using a screen-based Tobii Pro X2-30 eye tracker with a sampling rate of 30 Hz. The eye tracker was connected to the acquisition computer, which ran Tobii Studio 3.4.8 and collected eye-tracking data from the eye-tracking device. Using Tobii Studio 3.4.8, the areas of interest (AOIs) associated with the advertisements and further relevant elements of the user experience (e.g., chat, facecam, see Appendix A) were drawn. 

The Tobii I-VT fixation filter was used in Tobii Studio 3.4.8 to clean data according to the general procedure suggested by previous studies [71,72] before retrieving the fixation count associated with each AOI. The identification-velocity threshold (I-VT) fixation filter, an interpolation method aimed to fill in the gaps between the gaze data (maximum gap length 75 ms), was employed after the rejection of noise associated with tremors or micro-saccades. Moreover, saccades were rejected (fixations exceeding the angular velocity of 30°/s), brief adjacent fixations were combined (maximum angle between fixations set to 0.5° and maximum time between fixations set to 75 ms) into one, and fixations with a duration shorter than 60ms were excluded from the analysis. Finally, the visual attention associated with each AOI was quantified by considering the fixation count [73,74,75,76] divided by the total number of fixations that fell in the user screen during the total duration of the stimulus of interest and then expressed as a percentage [77].

### 2.4. Performed Analysis

Concerning RQ1, the visual attention associated with the facecam and chat was calculated through the average of the visual attention associated with the same AOIs across all videos, while the global visual attention related to IGAs was first obtained through the sum of the visual attention related to each advertising element (Left Banner”, “Right Banner”, and “Digital Billboards”) within every single video and then through the average of such values across videos. The Friedman analysis [78], followed by the Wilcoxon signed-rank test [79], was employed to compare the visual attention generated by the IGAs and the visual attention associated with other key elements of the game viewing experience (chat and facecam). The same procedure was employed to compare the visual attention associated with the “Left Banner”, “Right Banner”, and “Digital Billboards”.

With RQ2 focused on the comparison of the visual attention elicited by different ad formats (animated vs. static), the visual attention associated with the “Left Banner” was obtained through the average of the visual attention associated with the same AOI, respectively, across videos containing its “Animated” and “Static” format. The authors remind the reader that, due to the nature of the stimuli employed, the “Left Banner” represented the only AOI where such investigation could be performed (the “Right Banner” was always static, while the “Digital Billboards” were always animated). The Wilcoxon signed-rank test was employed to compare the visual attention elicited by the animated “Left Banner” and the visual attention elicited by the “Left Banner” when shown in a static format.

According to the objectives defined in RQ3, the visual attention associated with each IGA in the “Goal” and “No-Goal” scenes was computed through the average of the visual attention associated with the same AOIs across videos the contained respective “Goal” and “No-Goal” scenes. Afterward, the Wilcoxon signed-rank test was employed to verify, per each AOI (Left Banner”, “Right Banner”, and “Digital Billboards”), if the amount of visual attention was equally distributed across “Goal” and “No-Goal” scenes. 

**Figure A2:**
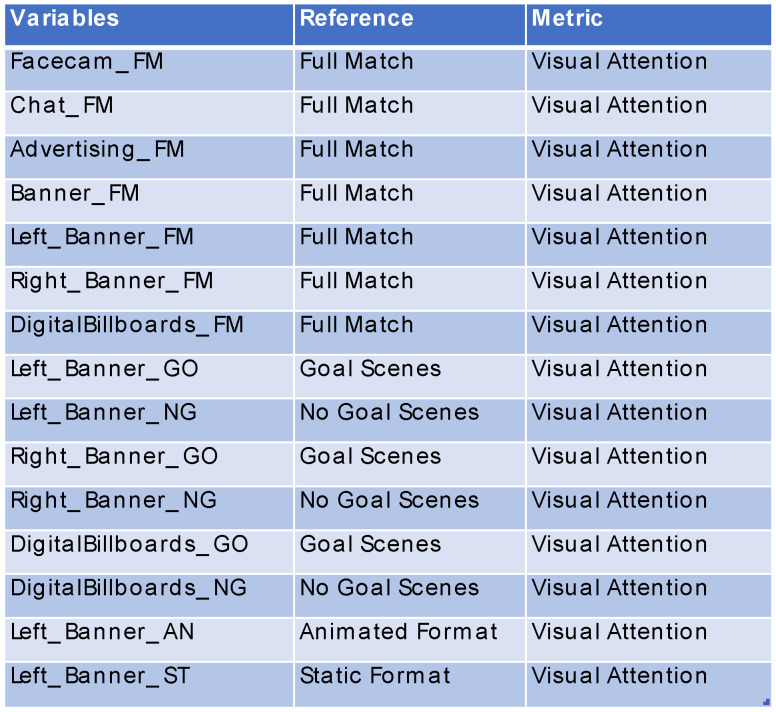
Variables selected for the study.

According to RQ1, RQ2, and RQ3, the performed analysis focused on the variables represented in Figure A2 (see Appendix A).

The non-parametric Friedman [78] and Wilcoxon [79] tests were performed to answer the aforementioned research questions since the obtained distributions were not normally distributed.

## 3. Results

### 3.1. IGAs, Facecam and Chat

**Figure 1:**
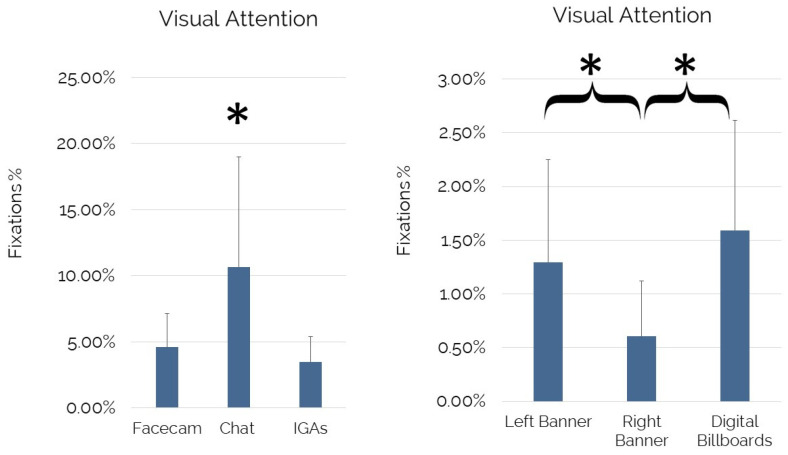
Visual attention for IGAs, Facecam, and Chat. Error bars represent standard deviation. Asterisks indicate statistically significant differences.

The Friedman analysis showed a significant effect of facecam, chat, and IGAs on visual attention (x^2^ = 26.692; *p* = 0.000). In particular, the Wilcoxon signed-rank test revealed a significant increase in visual attention for the chat in comparison to the facecam (Z = −4.85, *p* = 0.000, r = −0.50) and IGAs (Z = −4.80, *p* = 0.000, r = −0.50), and a significant increase in visual attention for the facecam in comparison to IGAs (Z = −2.39, *p* = 0.017, r = −0.25). Concerning the comparison within the IGAs, the Friedman analysis showed a significant effect of the IGAs on visual attention (x^2^ = 36.530; *p* = 0.000). In particular, the Wilcoxon signed-rank test revealed a significant increase in visual attention for “Left Banner” in comparison to “Right Banner” (Z = −4.65, *p* = 0.000, r = −0.48), and a significant increase in visual attention for the “Digital Billboards” in comparison to “Right Banner” (Z = −5.08, *p* = 0.000, r = −0.52). No statistical differences were detected while comparing the visual attention elicited by the “Left Banner” and the “Digital Billboards” (*p* = 0.135) (see Figure 1).

### 3.2. Left Banner in Animated and Static Format

**Figure 2:**
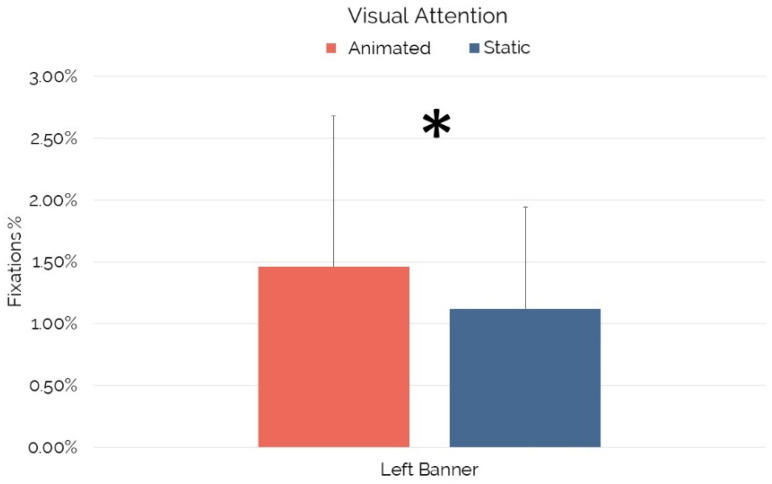
Visual attention for Left Banner in Animated and Static format. Error bars represent standard deviation. Asterisks indicate statistically significant differences.

Concerning the investigation performed on the “Left Banner”, which was available in static or animated format, the Wilcoxon signed-rank test revealed a significant increase in visual attention for the animated “Left Banner” in comparison to the “Left Banner” shown in static format (Z = −2.658, *p* = 0.007, r = −0.28) (see Figure 2).

### 3.3. IGAs in “Goal” and “No-Goal” Scenes

**Figure 3:**
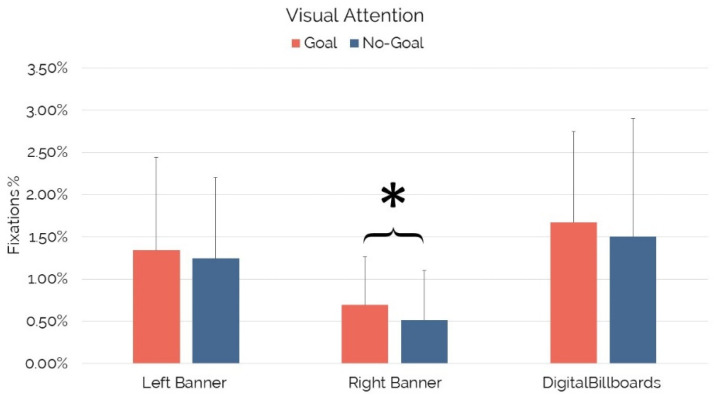
Visual attention for IGAs in “Goal” and “No-Goal” scenes. Error bars represent standard deviation. Asterisks indicate statistically significant differences.

According to the type of scenes shown, the Wilcoxon signed-rank test revealed that visual attention significantly increased for the “Right Banner” in “Goal” scenes in comparison to the “Right Banner” in “No-Goal” scenes (Z = −2.658, *p* = 0.008, r = −0.27). No statistical differences were detected while comparing the visual attention elicited by the “Left Banner” (*p* = 0.568) or the “Digital Billboards” (*p* = 0.095) in the “Goal” and “No-Goal” scenes (see Figure 3).

## 4. Discussion

### 4.1. Summary

With an estimation of 495 million people who tuned in to watch esports around the world (223 million esports fans and 272 million occasional viewers), sponsorships and media rights accounted for the majority of the USD 950.3 million in revenue generated by the global esports economy in 2020, an increase of 7.5% over the USD 584.1 million generated in 2019 [6]. In terms of peak internet traffic, Twitch, purchased by Amazon for USD 1 billion in August 2014, is only outperformed by Netflix, Apple, and Google, with the United States accounting for 20% of Twitch’s total traffic [80]. Despite such exponential growth, current scientific literature lacks evidence on how users pay attention to advertising stimuli while watching a virtual match provided through a streaming platform like Twitch, leaving companies partially unaware of the effectiveness of their investments.

To overcome such limitations, the authors relied on an eye-tracking research protocol that guaranteed the objective evaluation and quantification of the users’ visual attention through the collection of highly calibrated and cleaned gaze data [71,72] and the extraction of the Visual Attention metric [77]. This latter has the advantage of quantifying the visual attention in percentage, maintaining; therefore, a strong connection with the context of the study and with the total amount of visual attention available during the entire task (i.e., video observation).

As evidence of the aforementioned aspects, this study showed that IGAs were capable, altogether, of attracting, on average, 3.49% of the total visual attention available during the game viewing experience. Other key elements of the game viewing experience, such as facecam and chat, elicited a higher amount of visual attention than IGAs, with the chat drawing the highest amount of visual attention (10.68%). Unexpectedly, the results of the facecam (4.60%), although positive (+1.11% concerning IGAs), are not consistent with the widely supported concept of the human face as a more powerful attentional driver than other visual stimuli [81,82,83,84,85,86], thus opening new research avenues in contexts such as esports. In addition, this paper contributed to identifying the advertising spaces that generated higher visual attention during the game viewing experience, represented by the “Digital Billboards” (1.59%) and the “Left Banner” (1.30%). It is likely that the low performance of the “Right Banner” (0.61%) could be ascribed to its positioning and static nature, which needs further investigation. 

It is worth noting that the authors’ findings revealed the high eye-catching power associated with digital billboards in a context that entirely lacked evidence, disclosing new investment opportunities. Indeed, this is the first attempt to investigate the effectiveness of digital billboards in terms of visual attention, probably due to the complex procedure underlying such an objective. The visual attention related to digital billboards requires indeed a high effort during the AOI’s tracking process due to the continuous changes in shape and location of the digital billboards’ AOIs that varied according to the game camera’s movement on the X, Y, and Z dimensions (“Z”, in this context, is related to the zoom of the camera). This approach is featured by multiple adjustments during AOI’s tracking process that are not necessary for the in-game banner advertising tracking because the banners are located in the overlay, in a predefined area not affected by the game camera’s movements.

Another issue that has been addressed in the current study refers to the effectiveness that a given ad’s format can play in terms of visual attention. In particular, that participants were exposed during the game viewing experience to the “Left Banner” in two different formats: “Animated” and “Static.” Results showed that the “Left Banner” drew significantly higher visual attention when provided in animated format (1.46%) in comparison to the static format (1.12%). Such a finding provides evidence about the effectiveness of the animated format in esports contexts, assisting managers in evaluating future investments. Moreover, it provides additional insights into the long debate related to the efficacy of animated and static ad formats [56]. 

According to the limited-capacity model of attention [61,62] and a recent study [30], the authors investigated the amount of visual attention drawn by in-game advertising in “Goal” and “No-Goal” scenes, expecting higher visual attention toward IGA in “Goal” scenes, where the “battle” is steadily interrupted by specific events (i.e., celebration after scoring) in comparison to “No-Goal” scenes. This paper’s findings confirm prior research [30], although only concerning specific banner placements. In particular, the “Right Banner” elicited higher visual attention during “Goal” scenes (0.69%) in comparison to “No-Goal” scenes (0.51%), while the statistical significance was not reached for the other IGAs. This implies that the ad’s specificity (i.e., “Right Banner”) could play a crucial role in the distribution of visual attention in the “Goal” and “No-Goal” scenes. In addition, despite the “battle interruption” associated with the “Goal” scenes, it is worth mentioning that these latter include the most engaging scenes of a soccer match [67], which may therefore demand a higher level of visual attention [62,64] and mitigate the differences with the “No-Goal” scenes. Although further research is needed to clarify those aspects, such a first attempt of investigating the distribution of visual attention toward the ads in the “Goal” and “No-Goal” scenes may lead to the development of new pricing strategies related to the sales of IGA spaces, based on the real gaze experience of users and not on an arbitrary measure [46], and taking into account the different opportunities provided by distinct key scenes.

From a managerial perspective, this study highlights the advantages of accurately measuring the visual attention associated with the advertising elements during the game viewing experience. Since visual attention toward advertising is strongly related to industry revenues, managers should be aware of the level of attention elicited by different types of IGAs that could lead to new pricing strategies. As well, the various key elements of the streaming platforms (e.g., the streamer and the chat) need to be considered and evaluated in light of their attentional power, featured by both social and entertaining cues, to perfectly balance the elements that contribute to the game experience and the advertising success. The opportunity of assessing the visual attention in percentage, highlighting the key role of the context, strongly facilitates managers and experts operating in the advertising industry while reading and interpreting results based on eye-tracking data that reflect the real user’s gaze behavior.

### 4.2. Recommendations for Future Experimental Studies

Some limitations of the study and recommendations for future studies are discussed below.

The first limitation of the study is represented by the specificity of the context (i.e., specific ads and brands), which calls for additional studies with multiple matches, with different combinations in terms of brands, ads, match time duration, streamers, and chat. It is worth mentioning that the brands selected for the study were those included in the original FIFA20 match used as a reference (live streaming hosted on Twitch by a famous Italian pro player), a set of widely known global brands. In the near future, it might be interesting to also include additional brands that participants have no prior knowledge of (e.g., fake ads), to prevent any learning or retention effects.

Another limitation is related to the sample since it was composed only of men. Although this choice was made in light of the data associated with the esports industry, where women represent only 22% of esports fans [69], future research may verify if the findings provided by the authors could be extended to the female audience as well. 

Concerning eye-tracking measurement, our decision to focus on visual attention expressed as a percentage and based on fixation count was driven by the desire to maintain a strong connection with the context of interest and provide an approach that would aid advertising industry managers and experts in reading and interpreting eye-tracking results. Beyond that, it is worth mentioning that future eye-tracking research based on similar experimental protocols may provide further valuable evidence by exploring additional eye-tracking metrics such as fixation duration, time to first fixation (TTFF), or key events related to smooth pursuit eye movements. 

Moreover, it is worth noting that the sampling rate of the employed device was limited to 30 Hz. Although a low sampling frequency has been also used by similar studies [30,46], serving as a relevant source of inspiration for this work, some authors [87] still consider it sufficient to explore fixations as opposed to saccades. We believe that a higher sampling rate would have significantly increased the accuracy of our findings [88,89]. Another reason to increase the eye tracker’s sampling rate relies on the fact that, in recent years, new eye-tracking technologies, specifically designed for the gaming sectors and reaching high sampling rates (e.g., 133 Hz), have been introduced to the market [90].

We believe that the research protocol and results presented in this manuscript could inspire further research aimed to shed more light on the effectiveness of the chat and facecam and on the influence that such two key elements of the game-viewing experience may play on the entire context where they are embedded or on specific other elements (e.g., IGAs and their capacity of grabbing the users’ visual attention). For example, concerning the chat, a component that has been studied for decades [47,48] and that drew the highest amount of visual attention in the current study, it could be interesting to investigate to what extent the presence of chat emojis or different “chat update rates” may influence the attention toward the chat itself and/or toward other key elements (e.g., IGAs). The results obtained for the chat also showed that facecam, an element featured by social connectivity and interactivity [28,50,51], cannot be acknowledged as the most powerful attentional driver in this context, in contrast to other studies portraying the human face as the attention grabber par excellence [81,82,83,84,85,86]. To further explore such evidence, future research may explore if the facecam ability to capture the users’ attention changes over time (e.g., decreases) and to what extent such potential changes may affect the visual attention toward other key elements (e.g., IGAs). 

Finally, given the results related to the investigation of the visual attention elicited by ads in “Goal” and “No-Goal” scenes, future research could explore how both the interruptions (e.g., celebration after scoring) and the most arousing moments may affect the amount of attention captured by in-game advertising, leading to new insights and further managerial implications.

## 5. Conclusions

The current study aimed to shed more light on the perception of esports’ IGA in terms of visual attention, considering the exponential growth of the esports industry as well as the lack of scientific evidence in this context. 

At the same time, the authors demonstrated the potentiality of an advanced experimental protocol based on the eye-tracking technique. This latter, despite being designed specifically for the esports industry and related purposes, may be extended to the general advertising and gaming sector, particularly in those cases where it is crucial to quantify the visual attention taking into account the context where the stimuli of interest are embedded. 

The findings provided in this paper about the attentional-grabbing power of IGAs and other significant elements of the game viewing experience, the effectiveness of the different ad formats (e.g., static and animated), and key scenes (e.g., “Goal” and “No-Goal” scenes) may serve as a reference point for future research in the profitable esports sector, inspiring researchers and increasing the awareness of managers and advertisers concerning their investments and the design of new pricing strategies.

## Figures and Tables

**Figure A1:**
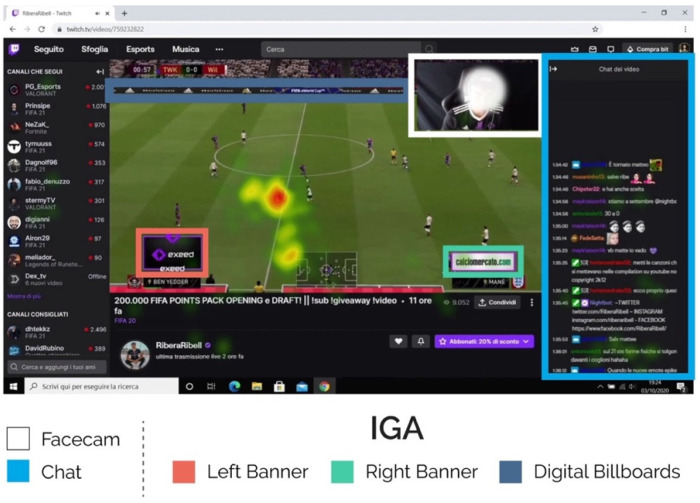
Areas of interest (AOIs) selected for the study. Note: The heat maps shown in the figure were not visible to the participants and were produced in the analysis phase.

## Data Availability

The data presented in this study are available on request from Prof. Babiloni (fabio.babiloni@brainsigns.com).
